# Refractory giardiasis in a child with steroid-resistant nephrotic syndrome

**DOI:** 10.1099/acmi.0.000476.v3

**Published:** 2023-04-25

**Authors:** Jitu Mani Kalita, Kirtika Sharma, Naila Mohammad, Vibhor Tak, Aliza Mittal, Vijaya Lakshmi Nag

**Affiliations:** ^1^​ Department of Microbiology, All India Institute of Medical Sciences, Jodhpur, Rajasthan, Pin-342005, India; ^2^​ Department of Pediatrics, All India Institute of Medical Sciences, Jodhpur, Rajasthan, Pin-342005, India

**Keywords:** *Giardia intestinalis*, nitroimidazole, nephrotic syndrome, corticosteroid

## Abstract

Giardiasis is an infection of the small intestine caused by the protozoan parasite *Giardia intestinalis* and one of the most common parasitic intestinal diseases in humans worldwide. It mainly manifests as a self-limited illness in the case of immunocompetent patients and usually does not require treatment. However, immunodeficiency is a risk factor for the onset of severe *Giardia* infection. In this report, a case of recurrent giardiasis refractory to nitroimidazole therapy is presented. A 7-year-old male patient with steroid-resistant nephrotic syndrome came to our hospital because of chronic diarrhoea. The patient was on long-term immunosuppressive therapy. Microscopic examination of stool showed a significant number of trophozoites and cysts of *G. intestinalis*. Treatment with metronidazole for longer duration than recommended has failed to clear the parasite in the present case.

## Data Summary

No data were generated in this case report.

## Introduction

Giardiasis is a parasitic infection caused by the flagellated protozoan *Giardia intestinalis*, also called *Giardia duodenalis* and *Giardia lamblia*, which colonizes the small intestinal lumen of vertebrate hosts. On the basis of broad range of host specificity, *Giardia* species have been divided into eight different assemblages (A to H). Assemblages A and B are exclusively associated with human infection, and other assemblages are mainly associated with infection of non-human species [1].

The majority of human cases take place in the developing world, where poverty and bad hygiene can lead to a childhood prevalence of up to 30 % [2]. A lower prevalence is observed in high-income countries, where infections are usually associated with travel. Infection occurs by ingestion of cysts in faecally contaminated food or water, or through person-to-person contact.

The clinical manifestations range from asymptomatic through common symptoms such as steatorrhoea and abdominal pain to systemic symptoms such as fever and weight loss. The diagnosis of giardiasis in a clinical setting is based on visual recognition of Giardia cysts or trophozoites by light microscopy, with or without concentration techniques.

Nitroimidazole derivatives are the first line of treatment for giardiasis and metronidazole and tinidazole are commonly used. Nitazoxanide is another option and, together with commonly used nitroimidazoles, the efficacy rates range from 60–100 % [3, 4]. However, nitroimidazole-refractory disease is increasingly reported and approximately 20 % of the individuals with giardiasis experience treatment failure after appropriate dose and duration [5].

Treatment failure for giardiasis is defined as the presence of the parasite in at least one of the infected patient’s three consecutive stool samples and the continuation of clinical symptoms even after the end of one or more sessions of recommended medication [6]. The attributable risk factors of treatment failures can be (i) reinfection, (ii) insufficient drug levels in the tissue, (iii) concurrent immunosuppression, (iv) drug resistance, (v) sequestration in the gall bladder or pancreatic ducts and (vi) unknown reasons [2, 6].

## Case report

A 7-year-old male child, with a known case of nephrotic syndrome under treatment, presented to paediatrics emergency in September 2019 with features of shock with abdominal swelling. The child also had a history of fever, multiple episodes of loose watery stools and vomiting (three–four episodes per day), along with dull abdominal pain prior to shock. He was admitted in paediatric intensive care unit and managed with antibiotics and inotropes.

Spontaneous bacterial peritonitis with sepsis was the diagnosis, although the aerobic culture of peritoneal fluid was negative. Blood and urine cultures were also sterile.

The child had nephrotic syndrome since the age of 1.5 years and was on prednisolone and steroid-resistant since December 2015. A renal biopsy was performed in January 2016 and reported minimal change disease.

The patient was then started on tacrolimus 1 mg twice daily orally. The first relapse after taking tacrolimus was reported in April 2017 followed by two more relapses in 2017. The dose of tacrolimus was increased to 1.2 mg in July 2017. After that, multiple episodes of relapse were seen. Up to January 2019 the patient was on 10 mg prednisolone once daily orally along with tacrolimus. In February 2019, the dose of tacrolimus was increased to 2 mg twice daily along with prednisolone 30 mg per day (1.5 mg kg^−1^).

During the course of treatment the child had developed steroid-induced posterior subcapsular cataract and grade II hypertension. For hypertension he was on antihypertensive medication. He also suffered from pulmonary tuberculosis in 2018, for which he completed treatment. Further, there was a history of failure to gain weight and height proportionate to age. On general examination, oedema was observed with rashes on the face and left knee.

**Fig. 1.**
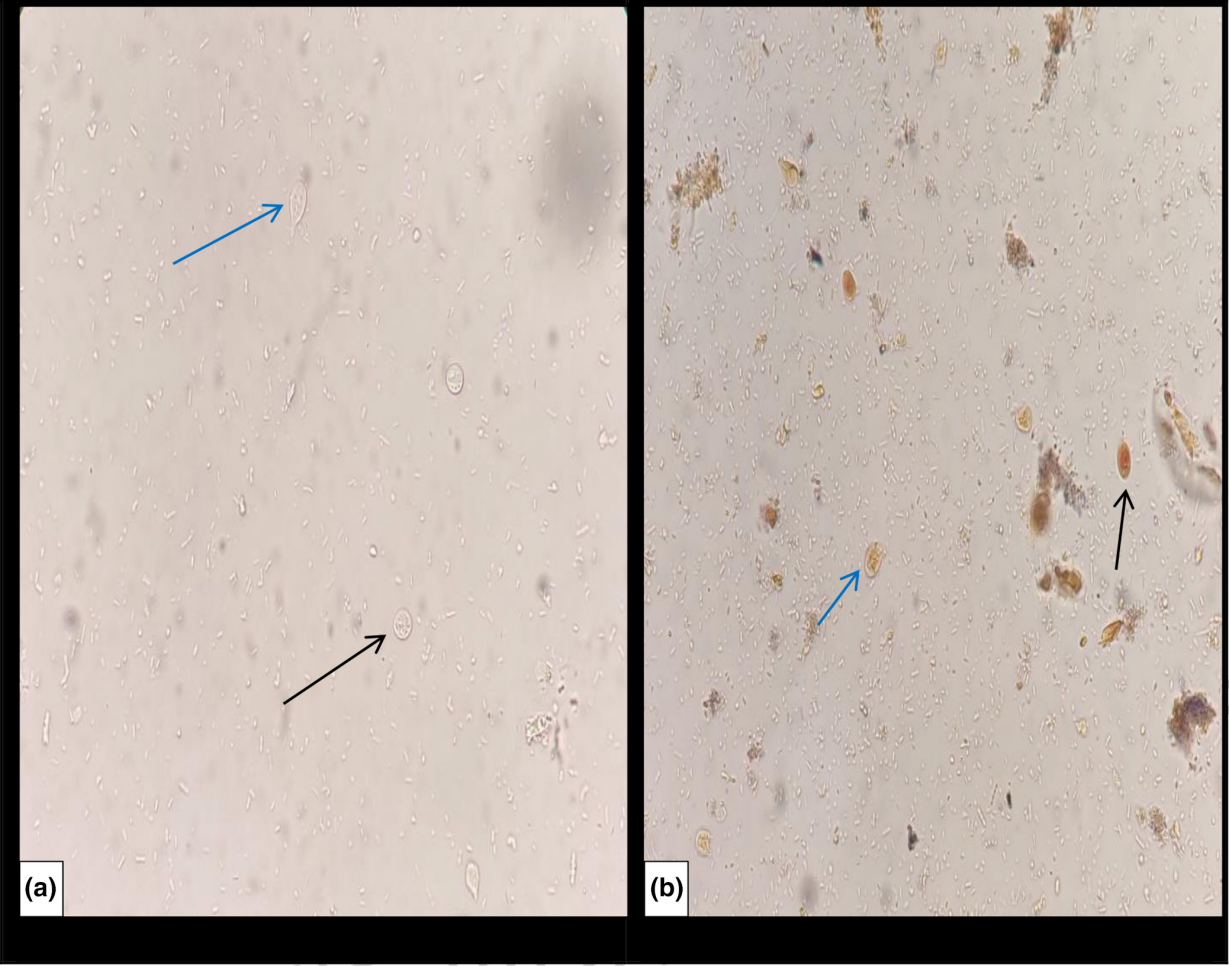
Cysts (black arrow) and trophozoites (blue arrow) of *Giardia intestinalis* in wet mount preparation: (a) saline and (b) iodine. Magnification, 400×.

**Fig. 2.**
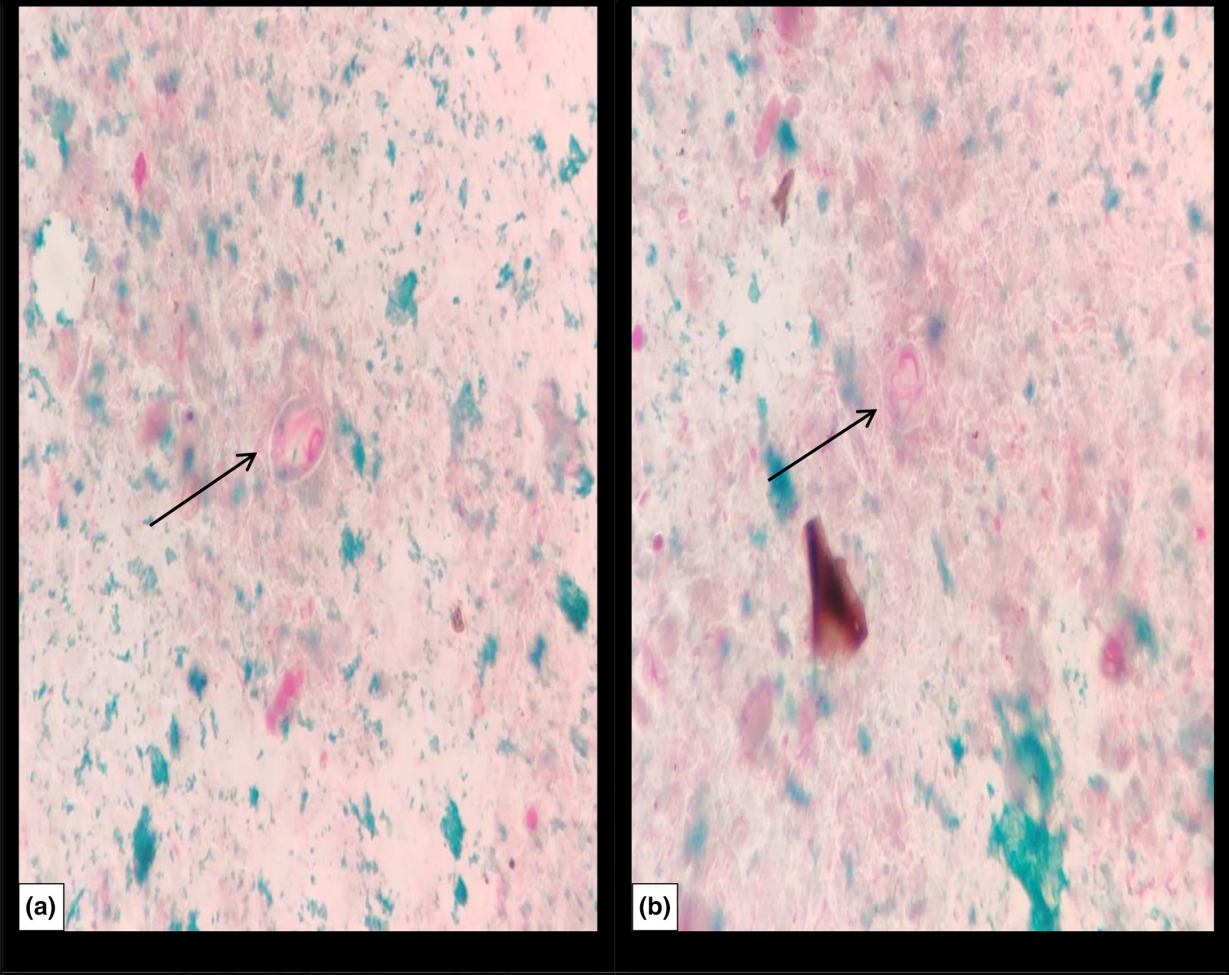
Chromotrope staining showing cysts (**a**) and trophozoites (**b**) of *Giardia intestinalis*. Magnification,1000×.

A stool sample was received in the microbiology laboratory and direct microscopy using 0.9 % saline and 1 % Lugol’s iodine wet mount preparations revealed trophozoites and cysts of *Giardia intestinalis*, shown in Fig. 1. Modified Ziehl–Neelsen staining was performed for the identification of coccidian parasites and microsporidia and was negative. Chromotrope staining was also performed to see microsporidia and was also negative. However, on chromotrope staining, lightly stained cysts and trophozoites of *G. intestinalis* were seen, as shown in Fig. 2. Stool culture was negative for enteric pathogen.

Metronidazole 100 mg three times daily orally was prescribed for 10 days. After completion of the therapy with metronidazole for 10 days, cysts and trophozoites of *G. intestinalis* were still detected in stool samples on multiple occasions (four occasions up to the first week of February 2020), along with persistence of clinical symptoms intermittently. The child was on and off metronidazole therapy for 4 months at the same dose. In the first week of February nitazoxanide 200 mg (twice daily orally) was added to the therapy for 7 days. The last stool sample received on last week of February 2020 was negative for *Giardia* cysts and trophozoite. The child also had a past history of passing loose stool on and off with increased frequency and bloating since for the previous 1.5 years, but no significant findings had been reported in stool samples previously.

## Discussion

*G. intestinalis* is a serious pathogen, especially for those who are immunocompromised or malnourished. Numerous studies have shown that immunocompromised persons are more likely to develop and manifest symptoms of *G. intestinalis* infections. Children with altered immunological responses, such as hypogammaglobulinaemia, common variable acquired immunodeficiency, nephrotic syndrome and protein–energy malnutrition, have been reported to have persistent giardiasis more frequently than other groups of children [7].

An Indian study by Yadav *et al*. showed a total 9.8 % cases of refractory giardiasis [1]. The rise in giardiasis susceptibility and the rate of therapeutic failure have traditionally been attributed to B cell dysfunction [8], but more recent research suggests that T cells may also play a role in these outcomes. In an adult mouse model, T cells were required to control *Giardia* infections [9], and evidence suggests that T cell dysfunction occurs in patients with common variable hypogammaglobulinaemia [10–12].

In this case, the patient was diagnosed with steroid-resistant nephrotic syndrome and was on prednisolone daily (for ~5 years) and tacrolimus daily for 3 years. The child had symptoms of chronic watery diarrhoea, abdominal cramps and weight loss. Microscopic examination of stool revealed a high number of *Giardia* trophozoites and cysts and could be the possible cause of hypovolemic shock in this patient.

In the present case, the patient was not responding to the standard treatment with metronidazole. Use of immunosuppressive drugs for longer periods in this patient could be the reason for this. Although treatment-refractory giardiasis is a problem in clinical practice, most of the refractory cases can be cured due to the large number of alternative and combination regimens of anti-giardial drugs. Apart from nitroimidazoles, the other drugs that can be used are albendazole, nitazoxanide, quinacrine and furazolidone [2]. In a study, combination of nitroimidazole and albendazole shows a cure rate of 60 % for patients with refractory giardiasis. Another study advised treating patients for 7 days with mepacrine (100 mg three times daily) if this combination proved unsuccessful, along with a warning about the possibility of developing acute psychosis [13].

In the present case, clearance of cysts and trophozoites of *Giardia* was observed microscopically after taking nitazoxanide for 7 days along with metronidazole. According to a recent systematic review [14], the preferred regimen for nitroimidazole refractory giardiasis are quinacrine for 5 days, albendazole plus nitroimidazole (metronidazole or tinidazole) for 7 days or mebendazole plus nitroimidazole (metronidazole or tinidazole) for 3 days. However, in this case nitazoxanide appeared to be effective along with metronidazole.

Although the child had experienced multiple episodes of gastroenteritis-like illness for a sustained period, no parasite had been detected on previous occasions. Low parasite level could be the reason for this, along with intermittent shedding of the parasite. A highly sensitive and specific enzyme immunoassay is available, which detects soluble *Giardia* cyst wall proteins and can be another option for diagnosis where microscopy is negative [15]. The results of this assay normally become negative when patients are treated effectively. In this case the child also had growth retardation and chronic giardiasis could have been an important contributor to this.

## Conclusion

Treatment-refractory giardiasis was documented by parasitological examinations in this case. Nitroimidazole-refractory giardiasis, along with other parasitic infections, can be a problem in clinical practice regarding patient management – as in this case – because corticosteroids are the mainstay of treatment. *In vitro* susceptibility testing for *Giardia* is difficult and genetic markers of drug resistance are also not known. There is no specific guideline for the empirical treatment of refractory disease. Starting with a combination of drugs can improve clinical outcomes in cases where there are predisposing factors such as immunosuppression, as in the present case.
